# Predicting outcome of Morris water maze test in vascular dementia mouse model with deep learning

**DOI:** 10.1371/journal.pone.0191708

**Published:** 2018-02-07

**Authors:** Akinori Higaki, Masaki Mogi, Jun Iwanami, Li-Juan Min, Hui-Yu Bai, Bao-Shuai Shan, Masayoshi Kukida, Harumi Kan-no, Shuntaro Ikeda, Jitsuo Higaki, Masatsugu Horiuchi

**Affiliations:** 1 Department of Molecular Cardiovascular Biology and Pharmacology, Ehime University, Graduate School of Medicine, Tohon, Ehime, Japan; 2 Department of Cardiology, Pulmonology, Hypertension and Nephrology, Ehime University, Graduate School of Medicine, Tohon, Ehime, Japan; 3 Department of Pharmacology, Ehime University, Graduate School of Medicine, Tohon, Ehime, Japan; Tokai University, JAPAN

## Abstract

The Morris water maze test (MWM) is one of the most popular and established behavioral tests to evaluate rodents’ spatial learning ability. The conventional training period is around 5 days, but there is no clear evidence or guidelines about the appropriate duration. In many cases, the final outcome of the MWM seems predicable from previous data and their trend. So, we assumed that if we can predict the final result with high accuracy, the experimental period could be shortened and the burden on testers reduced. An artificial neural network (ANN) is a useful modeling method for datasets that enables us to obtain an accurate mathematical model. Therefore, we constructed an ANN system to estimate the final outcome in MWM from the previously obtained 4 days of data in both normal mice and vascular dementia model mice. Ten-week-old male C57B1/6 mice (wild type, WT) were subjected to bilateral common carotid artery stenosis (WT-BCAS) or sham-operation (WT-sham). At 6 weeks after surgery, we evaluated their cognitive function with MWM. Mean escape latency was significantly longer in WT-BCAS than in WT-sham. All data were collected and used as training data and test data for the ANN system. We defined a multiple layer perceptron (MLP) as a prediction model using an open source framework for deep learning, Chainer. After a certain number of updates, we compared the predicted values and actual measured values with test data. A significant correlation coefficient was derived form the updated ANN model in both WT-sham and WT-BCAS. Next, we analyzed the predictive capability of human testers with the same datasets. There was no significant difference in the prediction accuracy between human testers and ANN models in both WT-sham and WT-BCAS. In conclusion, deep learning method with ANN could predict the final outcome in MWM from 4 days of data with high predictive accuracy in a vascular dementia model.

## Introduction

Dementia is becoming a serious health problem throughout the world, along with the aging of society. Since the importance of dementia study increases, the use of experimental methods to evaluate cognitive function is also increasing. The Morris water maze test is one of the most popular and established behavioral tests to evaluate rodents’ spatial learning and memory, which was originally invented by Richard G. Morris in 1983 [1]. This test has been widely used not only in the area of neuroscience but also in the field of cardiovascular study as the concept of the neurovascular unit became prevalent. However, its application for mice has some disadvantages, because this test was calculated for rats. First of all, the overall performance is lower in mice than in rats. In addition, their performance is susceptible to their swimming ability, vision and motivation. As a result, mice show floating and thigmotaxis more frequently than rats [2, 3]. This test also has some issues for testers. Although this method is a useful way to evaluate cognitive function, it requires a substantially longer experimental period. Considering the protocols used in recent studies, the standard training period in this test is about five days [4]. However, this period is not a fixed value and is left to the researcher’s discretion. In fact, the results of the final experimental day often seem self-evident to experienced testers. Therefore, we assumed that if the result on the final day can be estimated with high accuracy, it may be possible to shorten the experimental period and reduce physical and mental burden on testers.

Deep learning is a class of machine learning techniques which uses multiple layers of non-linear information processing to recognize feature quantities in data [5]. An artificial neural network (ANN) is one of the architecture to realize deep learning and has been applied for a variety of tasks including medical and pharmaceutical research [6, 7]. The major advantage of ANN is that we can easily obtain a highly accurate mathematical model from a certain amount of dataset without knowing the detailed internal process [8].

Therefore, we decided to construct an ANN system, in particular, a multiple layer perceptron (MLP), to estimate the final outcome in MWM from the previously obtained 4 days of data. To confirm the versatility of this system, we applied it to both normal mice and mice with impaired cognitive function. We selected a vascular dementia model with bilateral common carotid artery stenosis (BCAS) as the disease model because of its established method and protocol [9, 10].

After that, we also investigated the predictive accuracy of humans with the same datasets, and compared it with the result from the ANN system.

## Materials and methods

This study was performed in accordance with the National Institutes of Health guidelines for the use of experimental animals. All animal studies were reviewed and approved by the Animal Studies Committee of Ehime University. All of our raw data and script files were available from the online repository (https://www.protocols.io/private/c7e52000412cd34e2452fa893d35c617).

### Animals

Ten-week-old male C57B1/6 mice (Clea Japan Inc, Tokyo, Japan) as wild-type (WT) mice were enrolled in these experiments. The animals were housed in a room with a 12-hour light/dark cycle with a temperature of 25±1°C. They were given standard laboratory chow (MF; Oriental Yeast Co., Ltd., Tokyo, Japan) and water ad libitum. Some mice were used as an experimental disease model (n = 69) and the rest underwent sham-operation (n = 55).

### Bilateral common carotid artery stenosis (BCAS)

Among all 124 mice enrolled in this study, 69 mice underwent BCAS surgery at 10 weeks old. Micro-coils with an inner diameter of 0.18 mm, pitch 0.5 mm and total length 2.5 mm were used to create artificial stenoses in the bilateral common carotid arteries (CCAs). Before the procedure, mice were anesthetized with sodium pentobarbital (50 mg/kg intraperitoneal). Through a midline cervical incision, both CCAs were exposed and freed from their sheaths. The artery was gently lifted with a silk suture and then placed between the loops of a micro-coil. The micro-coil was twined around by rotating it around the CCA. Then another micro-coil was applied to the other CCA. After placing the coils, the incision was closed with sutures. Mice were placed gently on the soft carpet just after the procedure and kept warm until they recovered from anesthesia.

Samples with cerebral infarction were identified at the dissection stage and excluded from analysis. Cerebral infarction was detected by an apparent unilateral decrease of cerebral blood flow and/or apparent color change of the brain.

### Morris water maze test

MWM was performed 6 weeks after surgery (sham or BCAS), as described previously [11]. Mice were trained 5 times a day at 20-min intervals for 5 consecutive days. In each trial, mice were given 120 sec to find the platform. Swimming was video-tracked (AnyMaze; Stoelting Co., Wood Dale, IL), and the mean escape latency was recorded as a major outcome.

All obtained values were divided by 120 sec (maximus swimming time limit) in order to fall within the range between 0 and 1 so that they could be properly handled during ANN processing.

### Artificial neural network system

**Fig 1:**
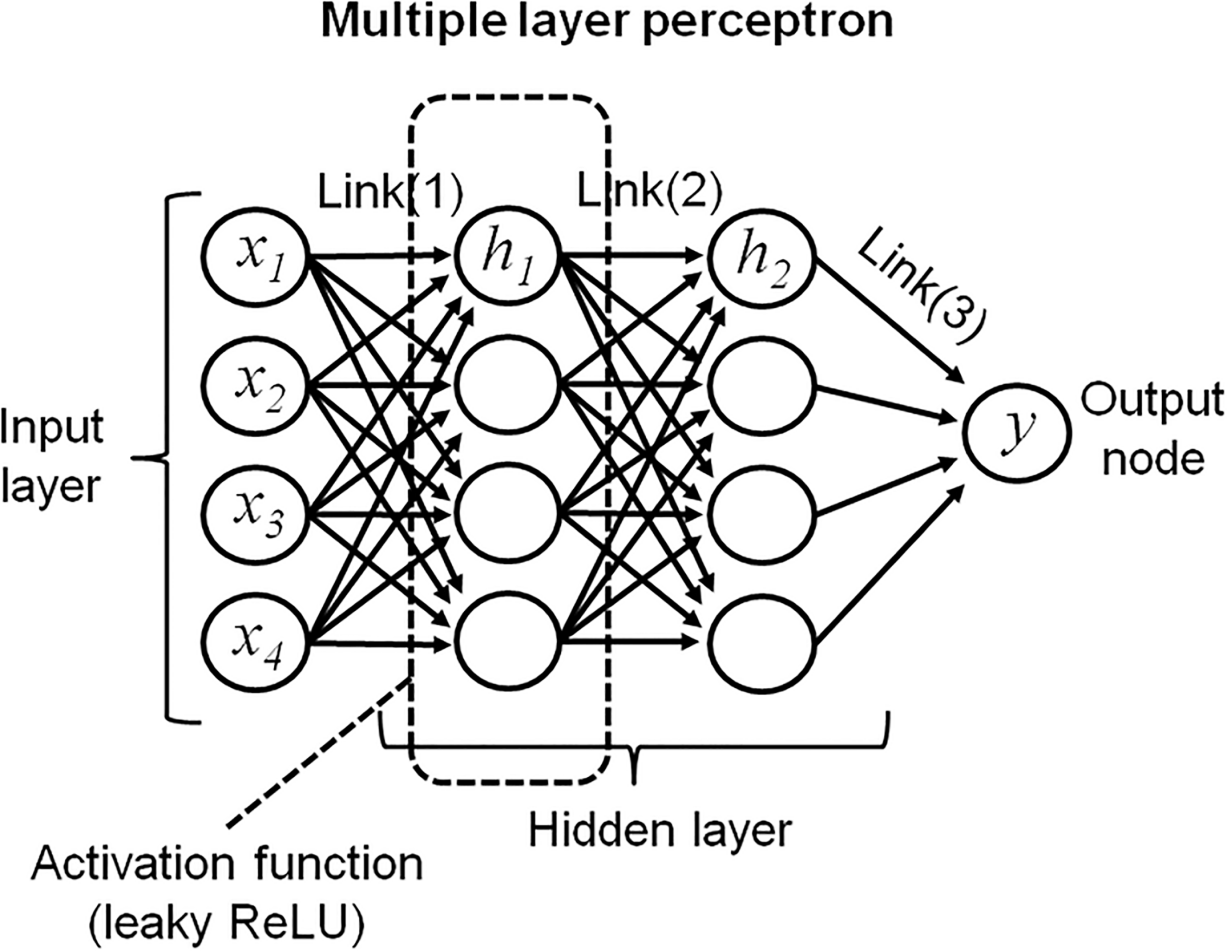
Schema of artificial neural network. The multiple layer perceptron is shown. The input layer consists of four nodes (*x*_1_-*x*_4_) which correspond to the values from day 1 to day 4. Each node is combined with a linear function (Link 1, 2 and 3) and transfers the value to the output node (*y*), which receives a value corresponding to the final outcome. The activation function is set to each node in the first hidden layer. ReLU, rectified linear unit.

An MLP with two hidden layers was used to predict the final outcome of the MWM. The structure of the ANN is shown in Fig 1. Each node in a layer was connected to the other layer with three linear combinations. The input layers sequentially imported 4 days of data and passed the values to the next nodes with linear transfer functions. We used a leaky rectified linear unit (ReLU) in the first hidden layer as an activation function. The value obtained from the output node was compared to the actual data on day 5, and the degree of error was calculated by least squares method, which is named “loss function”. Adaptive moment estimation (Adam) was selected as the optimization algorithm to minimize the loss function. Necessary gradients were calculated by backward propagation method (backpropagation). After a certain period of optimization with the training dataset, we could obtain the updated model to use in the validation stage.

K-fold cross-validation method was used for validation. The k value was empirically determined as five so that we can secure over 10 samples for validation. One data group out of five in each datasets was used for validation and the rest were used for training. Calculation was conducted five times for the different combinations and the averaged R-values were used for statistical analysis.

All these modeling processes were provided by Chainer, an open source framework for deep learning [12].

### Prediction by humans with experience

To evaluate the predictive accuracy of humans, we selected four researchers with sufficient experience in MWM from our laboratory. They were asked to predict the final outcome from the same dataset used for validation of the ANN system. Half number of the dataset was randomly selected from the original dataset and used for predictive task in each treatment group (WT-sham, n = 28; WT-BCAS, n = 35). Researchers were only given information on the mouse group (sham or BCAS treated), and predicted the actual measured value based on their intuition and experience.

### Statistical analysis

All data are presented as mean ± SEM. Data were analyzed with F-test followed by Welch’s t-test to assess the difference between two groups. Predictive accuracy was indicated as Pearson’s correlation coefficient. A value of P<0.05 was considered statistically significant.

## Results

### Comparison of mean escape latency between sham and BCAS treatment

**Fig 2:**
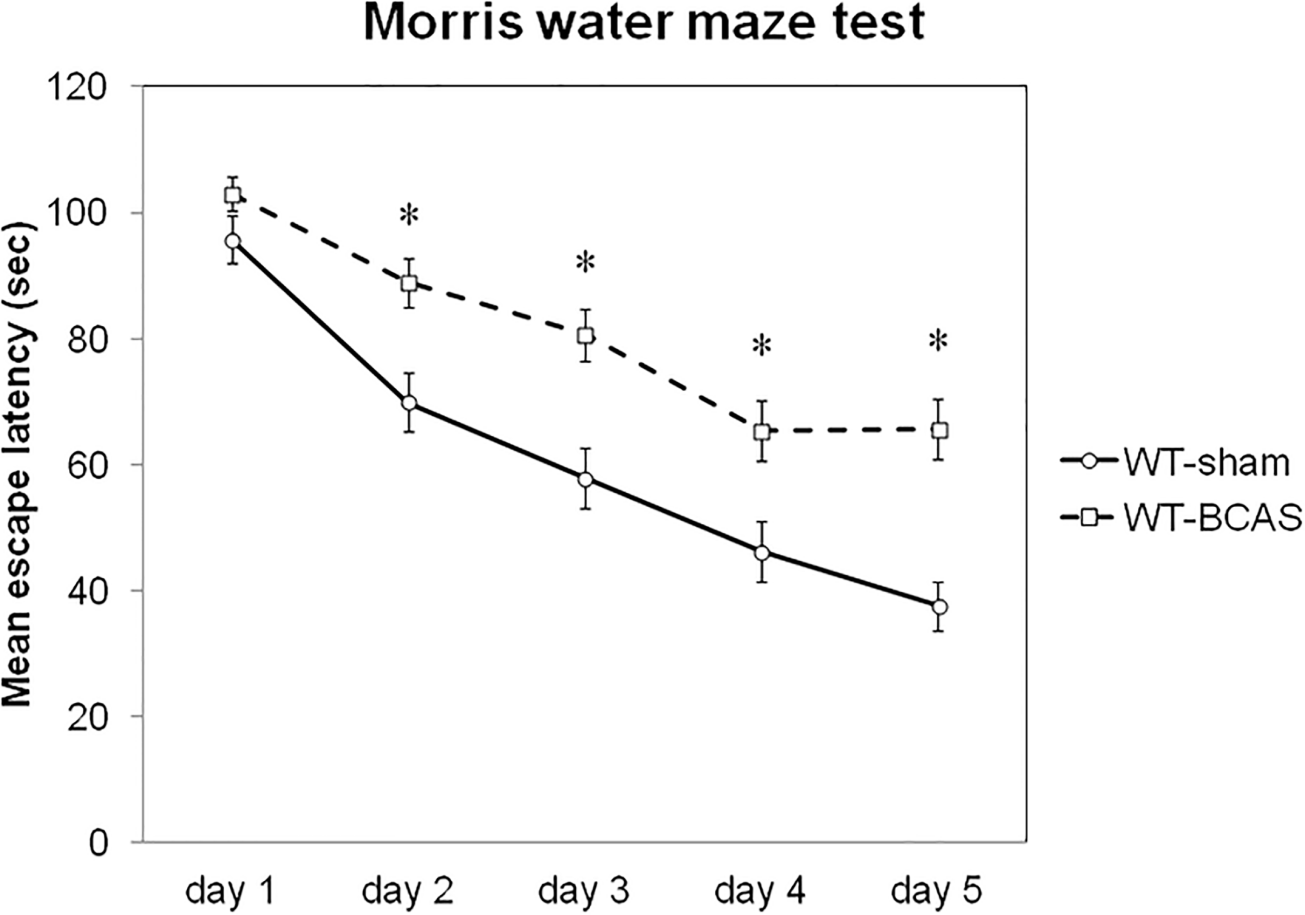
Mean escape latency in WT-sham and WT-BCAS. Mean escape latency was significantly longer in WT-BCAS from day 2 to day 5. WT, wild-type; BCAS, bilateral common carotid artery stenosis. *p<0.01 vs. WT-sham.

The time course of mean escape latency in all mice data is shown in Fig 2. Both WT-sham and WT-BCAS mice showed a reasonable learning curve. WT-BCAS mice showed significantly lower spatial learning ability compared to WT-sham from day 2 to day 5.

### Predictive accuracy of ANN system according to number of model updates

Table 1 shows the correlation between the predicted value with ANN and the actual measurement value in each group after 1000 epochs of model updates. There was a positive and strong correlation between the two values in both WT-sham and WT-BCAS. The model for WT-BCAS tended to show higher correlation coefficient compared to that for WT-sham (0.86 ± 0.04 vs 0.75 ± 0.03, p = 0.07).

**Table 1:** Predictive accuracy of ANN after 1000 epochs of model updates.

Treatment	Trial	Actual value	Predicted value	R-value	P-value
WT-sham	1	26.4 ± 4.7	29.1 ± 6.7	0.84	<0.01
	2	50.9 ± 9.1	39.5 ± 6.5	0.80	<0.01
	3	37.0 ± 4.3	43.8 ± 3.4	0.62	0.03
	4	53.8 ± 11.5	57.6 ± 7.4	0.74	<0.01
	5	18.8 ± 3.2	14.7 ± 2.8	0.73	<0.01
	Average	N/A	N/A	0.75 ± 0.03	N/A
WT-BCAS	1	43.5 ± 6.6	52.8 ± 7.9	0.77	<0.01
	2	68.5 ± 9.6	62.5 ± 9.0	0.78	<0.01
	3	71.0 ± 11.2	70.9 ± 8.7	0.90	<0.01
	4	73.7 ± 9.4	74.9 ± 10.4	0.88	<0.01
	5	69.9 ± 12.4	67.4 ± 10.1	0.98	<0.01
	Average	N/A	N/A	0.86 ± 0.04	N/A

R-value means Pearson’s correlation coefficient.

**Fig 3:**
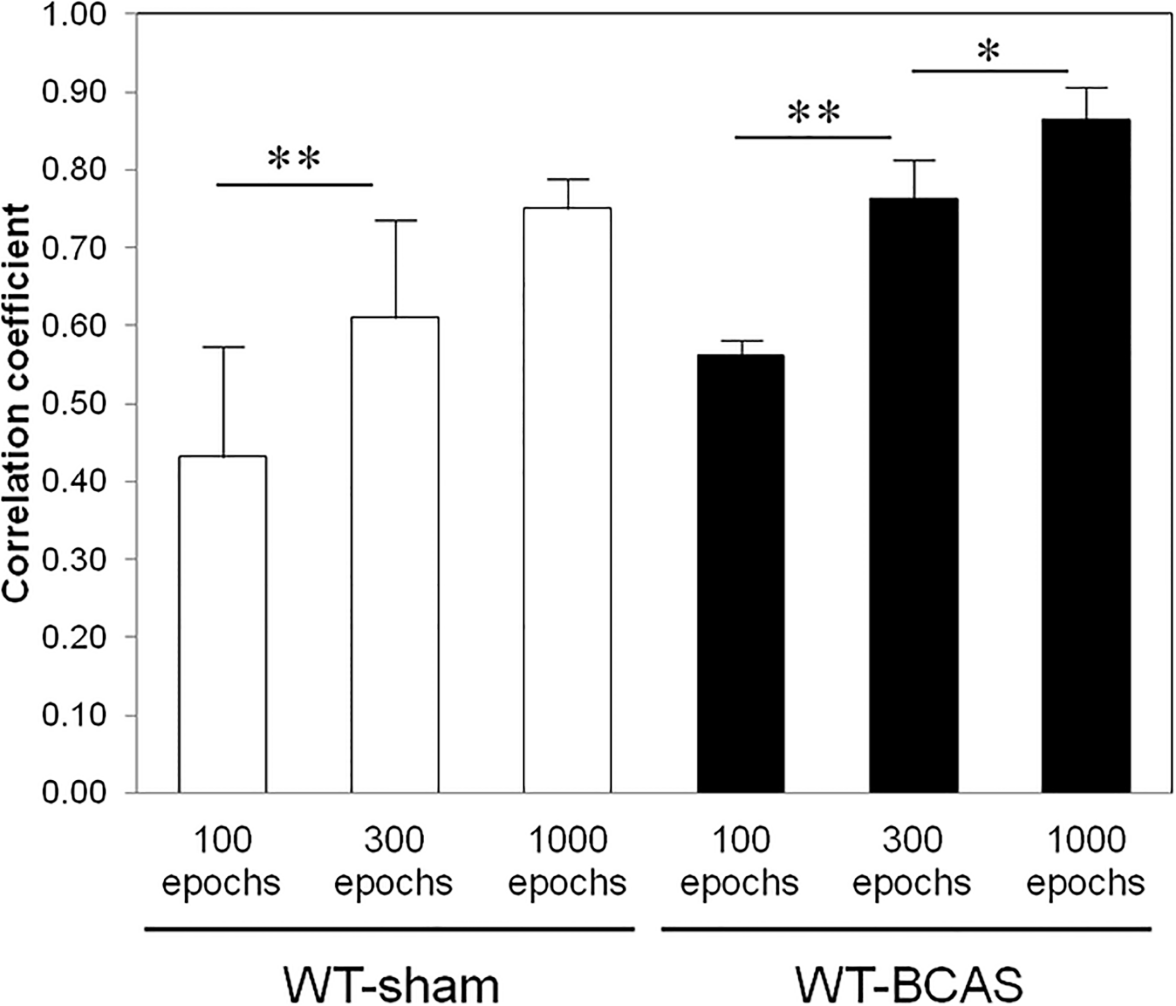
Predictive accuracy of ANNs according to the epoch numbers. Correlation between predicted value and actual measured value is shown for WT-sham and WT-BCAS according to different epoch numbers. R-value means Pearson’s correlation coefficient. *p<0.05, **p<0.01.

As the number of model optimizations increased from 100 to 1000 epochs, the correlation coefficient increased as well (Fig 3). The value of loss function reached a plateau after 1000 epochs of training, and no improvement of R-value was seen either (data not shown).

### Accuracy of human prediction

**Fig 4:**
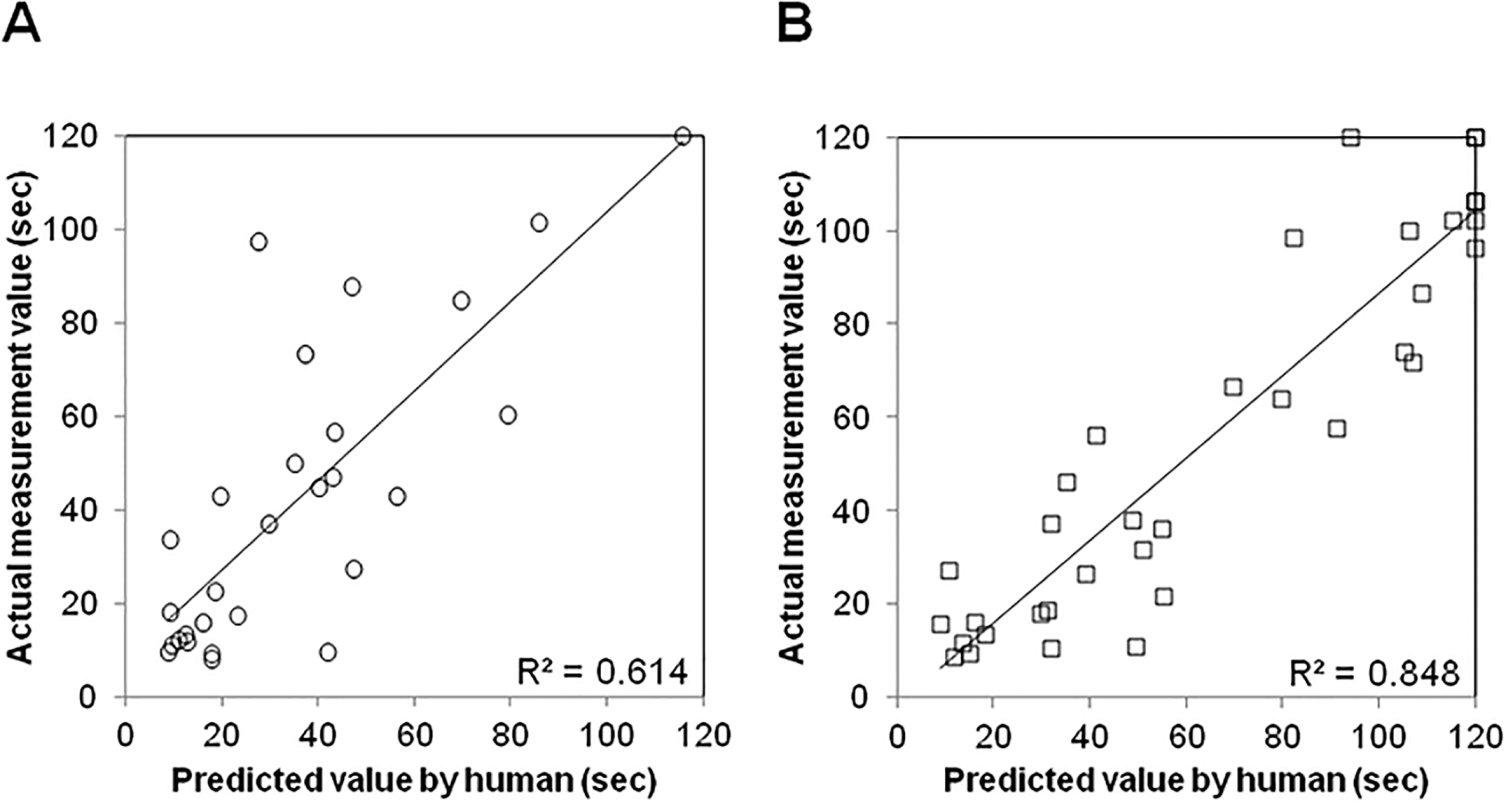
Correlation between actual value and predicted value by humans. Scatterplot between average predicted values and actual measured values is shown for WT-sham (A) and WT-BCAS (B) respectively. R^2^ means coefficient of determination.

**Fig 5:**
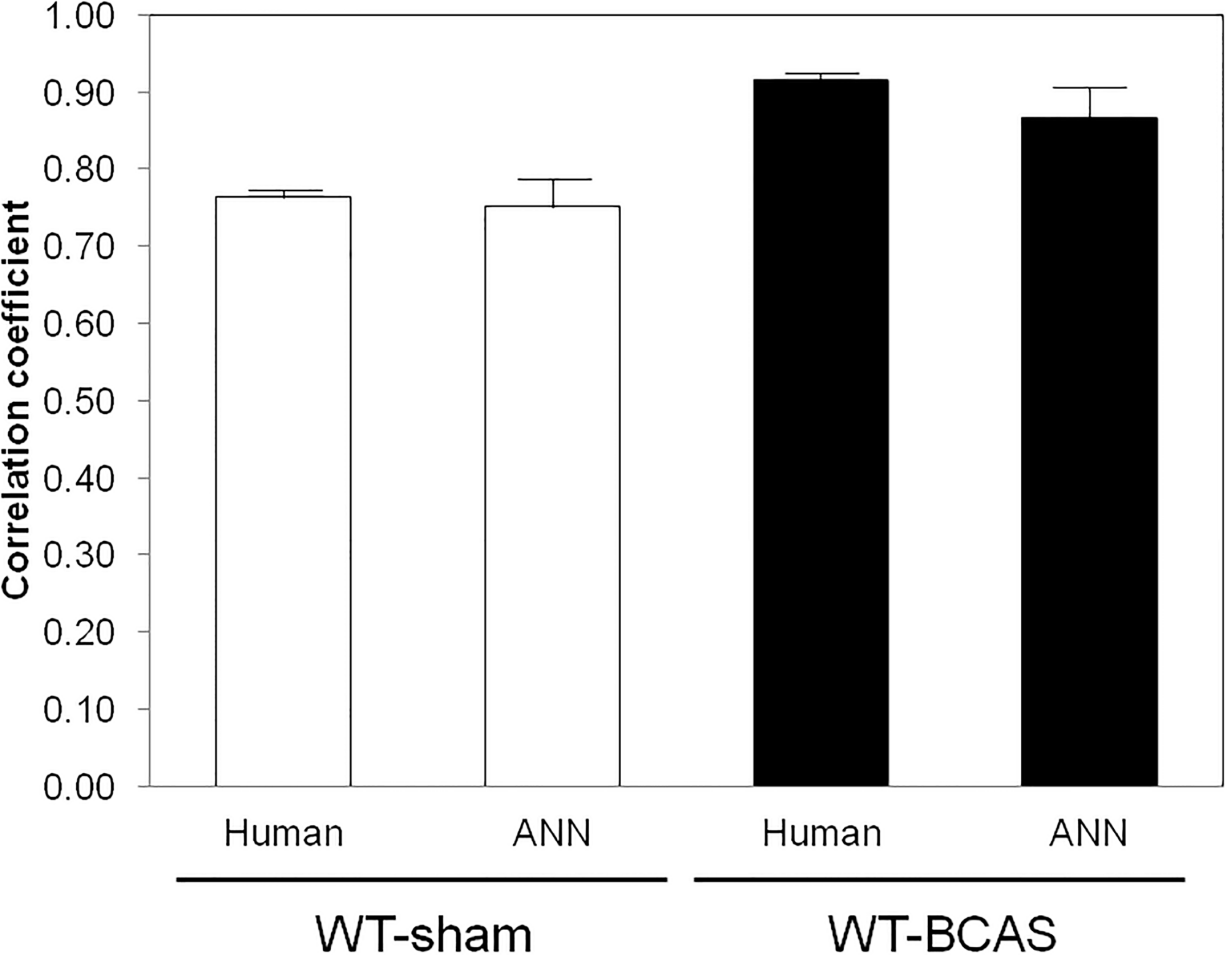
Comparison of predictive accuracy between human and ANN. The R-values between human prediction and that of ANN model were not significantly different in both WT-sham and WT-BCAS groups.

In order to compare the predictive accuracy between machine and human, we evaluated the ability of humans to estimate the experimental results based on their experience. Fig 4 shows the correlation between the actual value and the average predicted value of four researchers. The average predictive value, average actual value and their correlation coefficient according to the treatment groups were shown in Table 2. Similarly as in the case of ANN model, the R-value was significantly higher in WT-BCAS compared to that in WT-sham (0.91 ± 0.01 vs 0.76 ± 0.01, p<0.01). As shown in Fig 5, the accuracy of prediction was not significantly different from that of the ANN system in both WT-sham and WT-BCAS groups.

**Table 2:** Accuracy of human prediction.

Treatment	Subject	Actual value	Predicted value	R-value	P-value
WT-sham	1	35.1 ± 5.0	36.6 ± 6.2	0.75	<0.01
	2	35.1 ± 5.0	46.4 ± 6.8	0.76	<0.01
	3	35.1 ± 5.0	40.7 ± 6.4	0.73	<0.01
	4	35.1 ± 5.0	42.9 ± 6.2	0.80	<0.01
	Average	N/A	N/A	0.76 ± 0.01	N/A
WT-BCAS	1	64.9 ± 6.9	53.3 ± 6.4	0.89	<0.01
	2	64.9 ± 6.9	58.8 ± 6.6	0.93	<0.01
	3	64.9 ± 6.9	54.4 ± 6.6	0.90	<0.01
	4	64.9 ± 6.9	56.3 ± 6.9	0.92	<0.01
	Average	N/A	N/A	0.91 ± 0.01*	N/A

R-value means Pearson’s correlation coefficient.

*p<0.01 vs WT-sham.

## Discussion

ANN is a data-driven model and an effective tool for natural processes modeling. If a sufficient amount of information is given, it is possible to obtain an output value with high predictive accuracy without having explicit knowledge about the internal subprocess [13]. MLP is one of the most commonly used neural networks and has at least three layers of nodes.aft Since the advent of MLPs, ANNs have been able to recognize exclusive disjunction and have wide practical applications. Recent technical advancements such as some specific activation functions, application of backpropagation and the invention of autoencoder resolved the vanishing gradient problem and enabled the MLP to have deeper layers. Machine learning with such MLP with at least four layers has been called deep learning in a narrow sense [14]. Now this method is widely used, including for image diagnosis in clinical fields [15].

In contrast, the MWM is a way to evaluate the normality of a learning process. Rodents are forced to swim in a large round pool until they reach a platform hidden under the water, and the mean escape latency is measured. As the training progresses, mice learn the positional relation between the platform and the landscape and reach the goal in a shorter time. Thus, we can estimate their spatial learning ability from their mean escape latency. When mice are used, the standard training period is thought to be 5 days in general [1–4]. However, to our knowledge, there is no clear evidence that they require a 5-day training period. A recent study reported that cholinergic neuron activity in the hippocampal region significantly increased during the early stages of training [16]. Based on our experience, a mouse that shows good performance on the previous 4 days usually shows a better outcome on the final day. Similary, a mouse with a poor learning curve rarely shows a dramatic turnabout on the last day.

This suggests that we could possibly predict the final outcome if information on the past performance was available. Based on this concept, we made an ANN system to predict the outcome of MWM.

Since the experimental method is well established, we decided to employ a BCAS mouse model as subjects (n = 55) along with sham-operated WT mice (n = 69).

As a preliminary study, we tested the efficiency of two machine learning models using the all 124 mice data. In addition to the ANN model, we evaluated the predictive accuracy with support vector regression (SVR) model. Although it is generally conceived that SVR is more suitable to the time series prediction than ANN [17], there was no significant difference in the predictive accuracy between the two models in our study (Supporting Information).

Accordingly, we applied our ANN system to both groups and evaluated the efficacy respectively. Consistent with our previous report [9], WT-BCAS showed significant cognitive dysfunction compared to sham mice. This deterioration might be related to hippocampal neuronal dysfunction due to chronic cerebral hypoperfusion. According to the previous reports, BCAS treatment selectively impaired the working memory and did not affect the spatial learning ability in C57BL/6 mice [18, 19]. On the other hand, it is also reported that both working memory and reference memory were impaired in long-term BCAS model with hippocampal atrophy [20]. In our study, we assessed the cognitive function at later timing compared to the former two studies, so that we could detect the cognitive impairment with Morris water maze test.

The remote effect of pentobarbital on spatial learning is also suggested previously [21]. However, we did not see significant difference in spatial learning between WT-sham and control mice (without anesthesia or skin incision) in our preliminary experiment. We think this result is attributed to the difference in the timing of barbiturate exposure and the animal species.

An ANN system needs two types of datasets; one for training and the other for validation. The former datasets were used for optimization of MLP, and we derived the predicted value from the updated MLP with test data. We evaluated the correlation coefficient according to different numbers of cycles of optimization (epochs). When we increased the epoch number gradually from 100 to 1000, the error became smaller and Pearson’s R-value became higher. However, the value reached a plateau with over 1000 epochs of model optimization in both groups. Overfitting phenomenon is a common problem in an optimization process using backpropagation [22], but we did not experience such a situation in this model. This may because the escape latency graph generally shows a monotonic decrease and has a simple shape.

The correlation coefficient derived from ANN met the significance level in both WT-sham and WT-BCAS, but the R-value was higher in WT-BCAS in spite of the larger standard deviation (28.9 vs 40.5). This may be partly due to the relatively small number of WT-sham mice, but the loss of unexpected variance might be a feature of the homogenized pathological state in WT-BCAS.

Next, we analyzed the predictive capability of human testers to assess whether the ANN model has an advantage over the living brain. As a result, there was no significant difference in R-values between human prediction and the ANN system.

In conclusion, the outcome of MWM could be predicted from the previous 4 days of data with ANN, and the correlation coefficient was in no way inferior to that of experienced human testers.

In this study, we employed the most simple MLP model, so there should be room for improvement. We think the most important limitation is that both the explanatory variables and the objective variable are within the same data representation. Thus, other input parameters such as cerebral blood flow or blood pressure may raise the predictability of the model. Setting a retrieval test outcome as explanatory variable should be also a promising option in the future study.

We have to admit the study scope and the findings might be limited in terms of practical usage, but we still believe that trying to apply ANN for a variety of tasks is meaningful. We also think this study will stimulate discussion on the optimal training period in the Morris water maze test.

## Conclusion

An artificial neural network could predict the final outcome in MWM from 4 days of data with high predictive accuracy in a vascular dementia model.

## Supporting information

S1 Table Predictive accuracy in all mice with ANN. (PDF)

S2 Table Predictive accuracy in all mice with SVR. (PDF)

S3 Table Predictive accuracy of 3-day prediction model. (PDF)

S4 Table Accuracy of human prediction in 3-day task. (PDF)

S1 Fig Comparison of predictive accuracy in 3-day prediction task. The R-values between human prediction and that of ANN model were indicated. The R-value for WT-BCAS in human prediction was significantly higher than ANN.
(TIF)

S1 File Supplemental information. Additional study methods and results for the supporting information are described in detail.
(PDF)
