# Supplementary material for: Predicting outcome of Morris water maze test in vascular dementia mouse model with deep learning
Source: PLoS One. 2018 Feb 7;13(2):e0191708. doi: 10.1371/journal.pone.0191708 (PMC5802845; doi:10.1371/journal.pone.0191708)
Supplement: S1 Table — (PDF) [file pone.0191708.s001.pdf]

S1 Table. Predictive accuracy in all mice with ANN.

Trial	Actual value	Predicted value	R-value	P-value
1	37.9 ± 6.4	45.1 ± 7.0	0.87	<0.01
2	54.3 ± 6.7	54.7 ± 5.7	0.75	<0.01
3	56.3 ± 6.7	56.0 ± 6.3	0.83	<0.01
4	39.1 ± 6.8	42.1 ± 6.6	0.74	<0.01
5	74.5 ± 8.5	65.0 ± 6.2	0.95	<0.01
Average	N/A	N/A	0.83 ± 0.08	N/A

R-value means Pearson’s correlation coefficient.
