# Supplementary material for: Predicting outcome of Morris water maze test in vascular dementia mouse model with deep learning
Source: PLoS One. 2018 Feb 7;13(2):e0191708. doi: 10.1371/journal.pone.0191708 (PMC5802845; doi:10.1371/journal.pone.0191708)
Supplement: S2 Table — (PDF) [file pone.0191708.s002.pdf]

S2 Table. Predictive accuracy in all mice with SVR.

Trial	Actual value	Predicted value	R-value	P-value
1	37.9 ± 6.4	46.5 ± 6.1	0.88	<0.01
2	54.3 ± 6.7	53.3 ± 5.8	0.79	<0.01
3	56.3 ± 6.7	55.0 ± 5.9	0.80	<0.01
4	39.1 ± 6.8	42.8 ± 6.1	0.76	<0.01
5	74.5 ± 8.5	64.2 ± 6.7	0.94	<0.01
Average	N/A	N/A	0.83 ± 0.07	N/A

R-value means Pearson’s correlation coefficient.
