# Supplementary material for: Predicting outcome of Morris water maze test in vascular dementia mouse model with deep learning
Source: PLoS One. 2018 Feb 7;13(2):e0191708. doi: 10.1371/journal.pone.0191708 (PMC5802845; doi:10.1371/journal.pone.0191708)
Supplement: S3 Table — (PDF) [file pone.0191708.s003.pdf]

S3 Table. Predictive accuracy of 3-day prediction model.

Treatment	Trial	Actual value	Predicted value	R-value	P-value
WT-sham	1	26.4 ± 6.6	31.7 ± 5.1	0.75	<0.01
	2	50.9 ± 9.1	39.5 ± 6.7	0.44	0.16
	3	37.0 ± 4.3	40.9 ± 3.7	0.55	0.06
	4	53.8 ± 11.5	47.5 ± 4.2	0.5	0.12
	5	18.8 ± 3.2	30.9 ± 3.3	-0.14	0.68
	Average	N/A	N/A	0.42 ± 0.15	N/A
WT-BCAS	1	43.5 ± 6.6	60.3 ± 5.3	0.49	0.08
	2	68.5 ± 9.6	63.6 ± 8.1	0.53	0.06
	3	71.0 ± 11.2	63.1 ± 7.6	0.66	0.01
	4	73.7 ± 9.4	71.1 ± 5.0	0.63	0.02
	5	69.9 ± 12.4	72.9 ± 6.6	0.67	<0.01
	Average	N/A	N/A	0.60 ± 0.03	N/A

R-value means Pearson’s correlation coefficient.
