# Supplementary material for: Predicting outcome of Morris water maze test in vascular dementia mouse model with deep learning
Source: PLoS One. 2018 Feb 7;13(2):e0191708. doi: 10.1371/journal.pone.0191708 (PMC5802845; doi:10.1371/journal.pone.0191708)
Supplement: S4 Table — (PDF) [file pone.0191708.s004.pdf]

S4 Table. Accuracy of human prediction in 3-day task.

Treatment	Subject	Actual value	Predicted value	R-value	P-value
WT-sham	1	41.1 ± 5.9	41.1 ± 6.5	0.61	<0.01
	2	41.1 ± 5.9	48.7 ± 6.1	0.52	<0.01
	3	41.1 ± 5.9	38.8 ± 6.7	0.51	<0.01
	4	41.1 ± 5.9	39.8 ± 6.1	0.61	<0.01
	Average	N/A	N/A	0.58 ± 0.01	N/A
WT-BCAS	1	66.3 ± 7.0	67.3 ± 6.1	0.72	<0.01
	2	66.3 ± 7.0	73.5 ± 5.6	0.78	<0.01
	3	66.3 ± 7.0	65.3 ± 6.6	0.78	<0.01
	4	66.3 ± 7.0	64.3 ± 6.4	0.73	<0.01
	Average	N/A	N/A	0.73 ± 0.01*	N/A

R-value means Pearson’s correlation coefficient. *****p<0.01 vs WT-sham.
