# Supplementary figures and images for: Predicting outcome of Morris water maze test in vascular dementia mouse model with deep learning
Source: PLoS One. 2018 Feb 7;13(2):e0191708. doi: 10.1371/journal.pone.0191708 (PMC5802845; doi:10.1371/journal.pone.0191708)

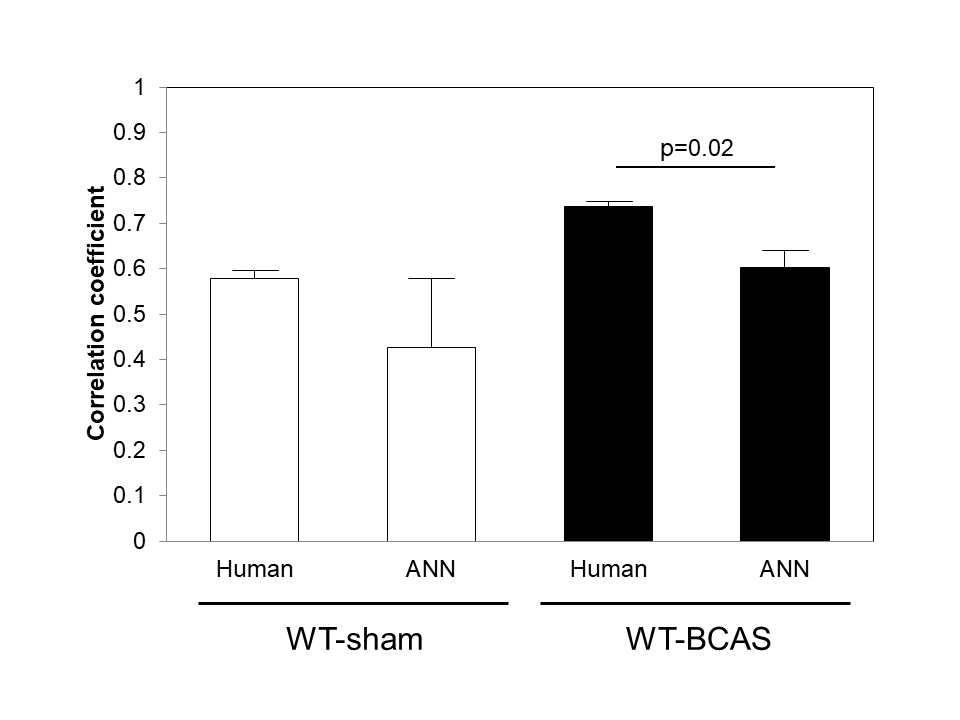

Supplement: S1 Fig — The R-values between human prediction and that of ANN model were indicated. The R-value for WT-BCAS in human prediction was significantly higher than ANN. (TIF) [file pone.0191708.s005.TIF]
