# Supplementary material for: Predicting outcome of Morris water maze test in vascular dementia mouse model with deep learning
Source: PLoS One. 2018 Feb 7;13(2):e0191708. doi: 10.1371/journal.pone.0191708 (PMC5802845; doi:10.1371/journal.pone.0191708)
Supplement: S1 File — Additional study methods and results for the supporting information are described in detail. (PDF) [file pone.0191708.s006.pdf]

Supplemental Information

Materials and Methods

This study was performed in accordance with the National Institutes of Health guidelines for the use of experimental animals. All animal studies were reviewed and approved by the Animal Studies Committee of Ehime University. All of our raw data and script files were available from the online repository (https://www.protocols.io/private/c7e52000412cd34e2452fa893d35c617).

Prediction for All Mice with Different Machine Learning Methods

We tested the predictive accuracy of artificial neural network (ANN) model in all mice regardless of BCAS treatment (n=124). In addition to the ANN model, we used support vector regression (SVR) model as another machine learning method. SVR model was constructed with 'scikit-learn', the open source machine learning library for Python. Optimal parameters for Gaussian kernel and regularization were determined by grid search. The performance of the model was evaluated with 5-fold cross-validation and the predictive accuracy was indicated as average R-values in each model.

3-day Prediction Model

We made an ANN model based on 3-day inputs (3-day prediction model). The ANN architecture was similar to that of 4-day prediction model. There are four layers including two hidden layers, but the each layer except output node was consisted of three nodes. The model's accuracy was also evaluated with 5-fold cross-validation.

Statistical Analysis

All data are presented as mean ± SEM. Data were analyzed with F-test followed by Welch's t-test to assess the difference between two groups. Predictive accuracy was indicated as Pearson's correlation coefficient. A value of $P<0.05$ was considered statistically significant.

Results

Predictive Accuracies in All Mice with Different Models

S1 Table shows the result with ANN model. We could see significant and strong correlation between the predictive values and the actual measured values and the average with ANN. The predictive accuracy with SVR model was indicated in S2 Table. The performance of SVR was similar to the ANN model and we could also see significant and strong correlation between the predictive values and the actual measured values.

Predictive accuracy in 3-day prediction model

As shown in S3 Table, we could not obtain significant correlation line in most trials of the machine learning and the correlation coefficients were lower than our previous model (WT-sham, $R=0.42 \pm 0.15$; WT-BCAS, $R=0.60 \pm 0.03$). We also evaluated the human's ability to predict the final outcome based on the 3-day data using the rest of the dataset which is used for 4-day prediction task. Human prediction's accuracy was also lower compared to 4-day prediction task shown in S4 Table (WT-sham, $R=0.68 \pm 0.02$; WT-BCAS, $R=0.74 \pm 0.01$). Similar to the 4-day prediction model, the R-value in WT-BCAS was significantly higher than in WT-sham ($p<0.01$). In addition, the average R-value was significantly lower in ANN model compared to human prediction in WT-BCAS group (shown in the S1 Fig).
